# Construction and characterization of a double mutant of *Enterococcus faecalis* that does not produce biogenic amines

**DOI:** 10.1038/s41598-019-53175-5

**Published:** 2019-11-14

**Authors:** Marta Perez, Marina Calles-Enríquez, Beatriz del Rio, Begoña Redruello, Anne de Jong, Oscar P. Kuipers, Jan Kok, M. Cruz Martin, Victor Ladero, Maria Fernandez, Miguel A. Alvarez

**Affiliations:** 1 Dairy Research Institute, Consejo Superior de Investigaciones Científicas (IPLA-CSIC), Paseo Rio Linares s/n, 33300 Villaviciosa, Spain; 2 Instituto de Investigación Sanitaria del Principado de Asturias (ISPA), Oviedo, Spain; 3 Department of Molecular Genetics, Groningen Biomolecular Sciences and Biotechnology Institute, University of Groningen, Nijenborgh 7, 9747 AG Groningen, The Netherlands

**Keywords:** Applied microbiology, Molecular biology, Applied microbiology, Molecular biology

## Abstract

*Enterococcus faecalis* is a lactic acid bacterium characterized by its tolerance of very diverse environmental conditions, a property that allows it to colonize many different habitats. This species can be found in food products, especially in fermented foods where it plays an important role as a biopreservative and influences the development of organoleptic characteristics. However, *E. faecalis* also produces the biogenic amines tyramine and putrescine. The consumption of food with high concentrations of these compounds can cause health problems. The present work reports the construction, via homologous recombination, of a double mutant of *E. faecalis* in which the clusters involved in tyramine and putrescine synthesis (which are located in different regions of the chromosome) are no longer present. Analyses showed the double mutant to grow and adhere to intestinal cells normally, and that the elimination of genes involved in the production of tyramine and putrescine has no effect on the expression of other genes.

## Introduction

*Enterococcus faecalis* is a Gram-positive bacterium of the phylum Firmicutes. It is tolerant of very diverse environmental conditions, which allows it to colonize many different habitats, including water, soil, the gastrointestinal tract (GIT) of different animals (from insects to mammals, including humans)^1^, and food products, especially fermented foods. *E. faecalis* is a member of the lactic acid bacteria (LAB), which are responsible for numerous food fermentations. These bacteria therefore play a role as biopreservatives. Unlike for most LAB, however, allowing the presence of *E. faecalis* in food is controversial. In artisanal cheeses, *E. faecalis* is believed involved in the ripening process via its proteolytic activity, and in the development of desirable aromas and flavours. It is also an important producer of bacteriocins with activity against pathogenic bacteria and food spoilage microorganisms, making it a potential biopreservative^2^. Some strains of *E. faecalis* are even considered to benefit human health^3^. For example, *E. faecalis* Symbioflor 1 has been marketed as a probiotic for more than 50 years^4^. However, some strains have virulence factors and are resistant to antibiotics, and have caused serious infections, especially in the hospital environment^5^. Indeed, vancomycin-resistant enterococci are regarded as a serious threat to human health by the World Health Organization. In fermented foods such as cheese, *E. faecalis* is also the bacterium largely responsible for the accumulation of tyramine and putrescine^6–8^, biogenic amines (BA) that can reach concentrations so high that they cause headaches, migraines and even hypertensive crises^9^. Putrescine and especially tyramine^10,11^ are also cytotoxic at concentrations easily reached in cheese. Furthermore, tyramine is genotoxic for intestinal cells *in vitro*; it might therefore play a role in the promotion of intestinal cancer^12^.

In *E. faecalis*, putrescine is made via the agmatine deiminase pathway, in which agmatine is deiminated to putrescine with the concomitant formation of CO_2_, ATP and ammonium ions^13^. This route provides energy to the cell in the form of ATP as well resistance to acid stress^14^. The genes involved in this pathway make up the *agdi* cluster; *aguD* codes for the agmatine/putrescine antiporter, *aguA* for agmatine deiminase, *aguB* for putrescine transcarbamylase, and *aguC* for a specific carbamate kinase. These genes are cotranscribed in a polycistronic mRNA, the formation of which is induced by the presence of agmatine (the substrate for the reaction), and via AguR, a transcription regulator encoded by *aguR* located upstream of *aguD* but with the opposite orientation^14,15^.

Tyramine is produced by the decarboxylation of the amino acid tyrosine, via the action of tyrosine decarboxylase (TdcA). Tyramine is secreted from the cytoplasm in exchange for tyrosine by the antiporter TyrP. This mechanism helps *E. faecalis* to adapt to acidic environments such as the stomach or fermented foods by maintaining its intracellular pH^16^. The proteins involved in this pathway are encoded in the *tdc* cluster, which contains four genes in the following order: *tyrS*, an aminoacyl transfer RNA (tRNA) synthetase-like gene; *tdcA*, which codes for the decarboxylase TdcA; *tyrP*, which encodes the antiporter TyrP; and *nhaC-2*, which codes for a protein thought to be an Na^+^/H^+^ antiporter, although its role in the synthesis of tyramine remains uncertain. This gene has been found in all the LAB *tdc* clusters so far examined^17^. *tyrS* is transcribed as monocistronic mRNA, while *tdcA*, *tyrP* and *nhaC-2* are co-transcribed as a polycistronic mRNA^16^.

It is generally believed that the synthesis of BA by LAB is a *strain* characteristic, and screening for non-BA producing strains with good biotechnological or probiotic traits is routinely performed^18,19^. *E. faecalis*, however, shows the *species*-specific trait of producing both tyramine and putrescine^13^; screening for non-BA producing strains for technological or biomedical uses is, therefore, not an option. The possibility of generating and screening large collections of spontaneous mutants has recently been suggested for finding those without the capacity to produce tyramine; strains with this characteristic might be of use in food fermentations^20^. Unfortunately, this is a long and laborious business and the result could be the finding of a non-BA-producing strain that has also lost desirable phenotypic characteristics naturally associated with BA production, such as good growth performance^21^. Thus the overall involvement of the BA formation pathways in the physiology of the target strain or species should be known before setting out to follow such a long and tedious strategy.

The aim of the present work was to construct, by homologous recombination, a double mutant of *E. faecalis* via the deletion of the *agdi* and *tdc* clusters, and to check how this affects the fitness of the bacterium and its capacity to colonize the GIT. In addition, the effect of the deletion of these clusters on the expression of other genes was examined using transcriptional microarrays.

## Results

### The proteins involved in the putrescine biosynthesis pathway of *E. faecalis* are encoded in the *agdi* cluster

The involvement of the *tdc* cluster genes in the synthesis of tyramine in *E. faecalis* was already known; our group reported how a mutant in which the *tdc* cluster had been deleted was unable to produce tyramine^16^. In the present work, the production of the *E. faecalis* V583 Δ*agdi* deletion strain (an intermediate in the production of the non-BA producer double mutant), confirmed the function of the putative *agdi* cluster (as identified by its similarity to the nucleotide sequence of this cluster in other LAB). Its inability to produce putrescine, as determined by UHPLC analysis of the cultures supplemented with agmatine (data not shown), revealed the genes of the *agdi* cluster of *E. faecalis* to be responsible for putrescine production.

Once this was confirmed, the *tdc* cluster was deleted in the *E. faecalis* V583 Δ*agdi* strain. The generated double deletion mutant was named *E. faecalis* V583 Δ*agdi*Δ*tdc*, and it was confirmed that this double mutant produced neither tyramine nor putrescine (data not shown).

### Putrescine and tyramine production slightly improves the growth of *E. faecalis*

**Figure 1:**
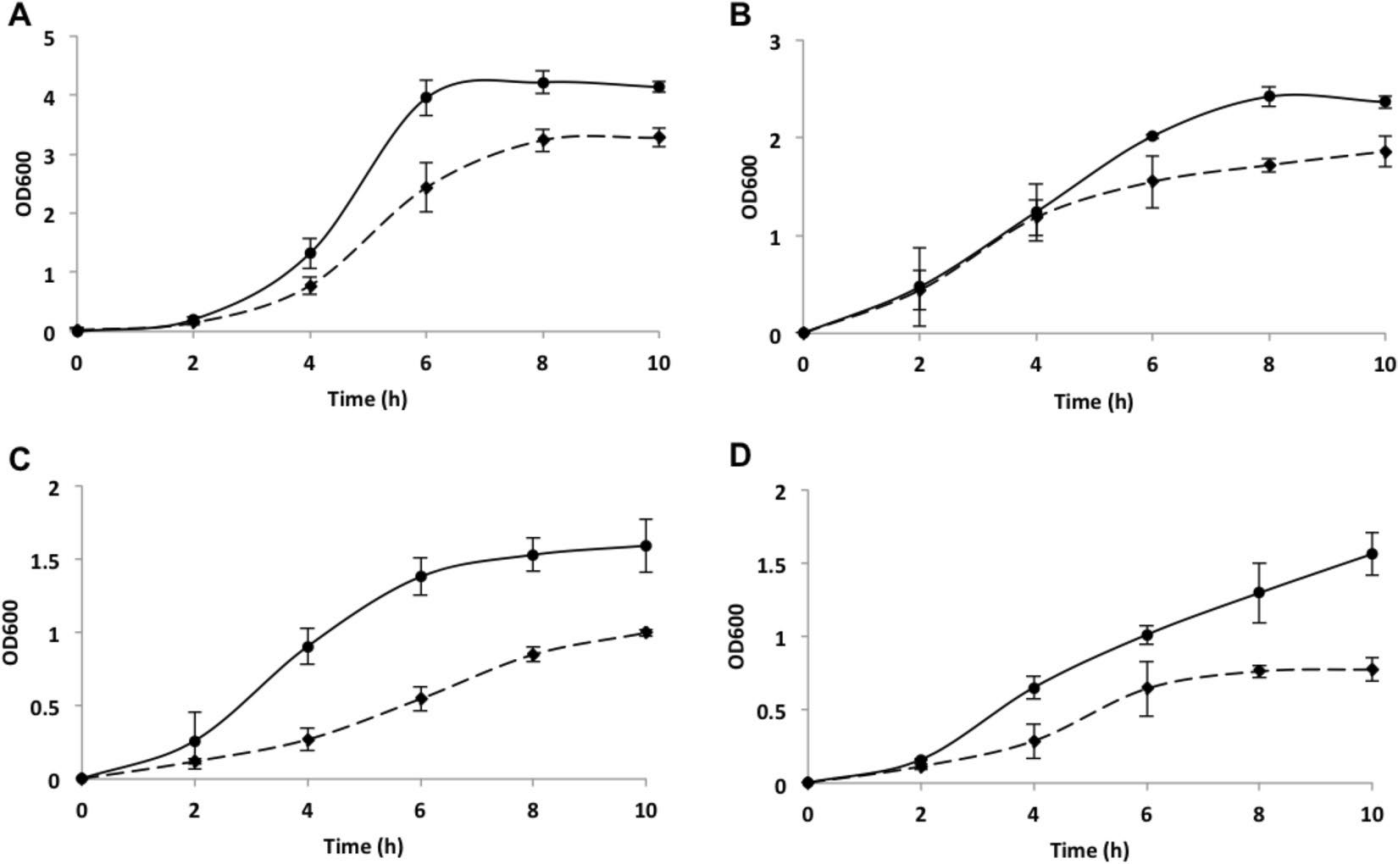
Growth curves for *E. faecalis* V583 (continuous line) and *E. faecalis* V583 Δ*agdi*Δ*tdc* (discontinuous line) in media supplemented with 20 mM agmatine and 10 mM tyrosine. (**A**) influence of putrescine and tyrosine synthesis on cultures grown in GM17. (**B**) effect of limiting the carbon source on cells cultured in M17 supplemented with glucose at 1 g L^−1^. (**C**) influence of acidic pH on the growth of strains propagated in GM17 adjusted to an initial pH of 5.0. (**D**) growth under a combination of carbon source depletion and acidic pH in M17 with glucose 1 g L^−1^ and pH adjusted to 5.0. The OD_600_ was monitored over 10 h.

The influence of the simultaneous production of putrescine and tyramine on the growth of *E. faecalis* was studied by monitoring the OD_600_ of wt and Δ*agdi*Δ*tdc* cultures in GM17 supplemented with agmatine and tyrosine. The mutant strain achieved a maximum OD_600_ of 3.3, while the wt strain reached a value of 4.1 (Fig. 1A). The effect of BA production was also determined under carbon source depletion by growing the strains in M17 with agmatine and tyrosine and a reduced concentration of glucose. Figure 1B shows the maximum OD_600_ achieved by both the wt and Δ*agdi*Δ*tdc* strains to be reduced compared to the standard carbon source condition (Fig. 1A). The mutant strain returned a reduced OD_600_ value (1.8 vs. 2.3), indicating a role for the BA pathways in growth under this stress condition.

The strains were then grown in GM17 with the same substrates but with the initial pH adjusted to 5 to examine the influence of the BA pathways in an acidic environment. While the wt strain reached an OD_600_ of 1.6, the mutant strain only managed an OD_600_ of 1 and showed a less steep exponential phase slope (Fig. 1C). Finally, the growth of the wt and Δ*agdi*Δ*tdc* strains was monitored in M17 with reduced glucose and an acidic initial pH in the presence of agmatine and tyrosine. The OD_600_ values obtained (Fig. 1D) were similar to those recorded for the acidic conditions (Fig. 1C).

These outcomes show that, despite the lack of BA biosynthesis, the Δ*agdi*Δ*tdc* mutant was still able to grow reasonably well.

### The mutant strain *E. faecalis* Δ*agdi*Δ*tdc* tolerates transit through an *in vitro* gastrointestinal tract model

**Figure 2:**
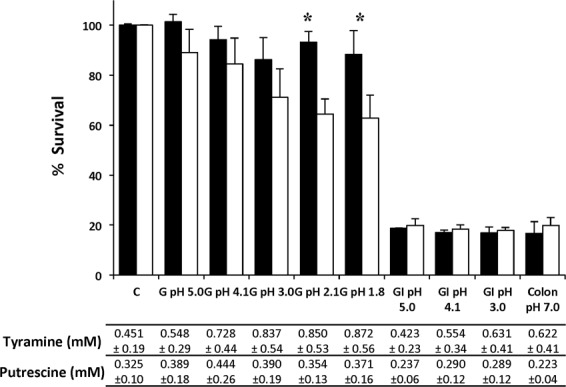
Gastrointestinal transit simulation for *E. faecalis* V583 (black bars) and *E. faecalis* V583 Δ*agdi*Δ*tdc* (white bars). Survival (%) of strains under gastric (G), gastrointestinal (GI) and colonic stresses in the presence of 20 mM agmatine and 10 mM tyrosine. C, untreated cells (control). Survival was measured using the LIVE/DEAD^®^ BacLight fluorescent stain kit. Values are expressed as a percentage of the control value. Cells from cultures grown with 20 mM agmatine and 10 mM tyrosine for 6 h were used in all cases. An asterisk indicates a significant difference (*p* < 0.05; Student *t* test). The putrescine and tyramine concentrations accumulated by the wt strain under each condition are indicated below the graph.

The wt and Δ*agdi*Δ*tdc* strains were challenged with GI conditions in the presence of agmatine and tyrosine (Fig. 2). Greater concentrations of tyramine were recorded in the more acidic gastric conditions (pH 3.0, 2.1 and 1.8), while greater putrescine accumulation was seen at pH 4.1.

The viability of the mutant strain was some 10–35% lower than for the wt strain under gastric stress conditions (Fig. 2). This reduction became significant at pH 2.1 and 1.8, coinciding with the conditions under which the wt strain accumulated tyramine more strongly. Under the conditions simulating the end phase of digestion in the colon, approximately 65% of the Δ*agdi*Δ*tdc* cells survived.

The survival of both the wt and Δ*agdi*Δ*tdc* populations was reduced to approximately 17% under the gastric and colonic stresses. Although the wt strain was able to synthesise both BA under both of these conditions, no significant differences between the strains were observed in terms of survival. These findings indicate that, although the mutant survived the acidic environment less well than did the wt strain, a significant proportion of the population can tolerate GI stress and reach the colon.

### The adhesion of *E. faecalis* to intestinal epithelial cells is not affected by the synthesis of biogenic amines

**Figure 3:**
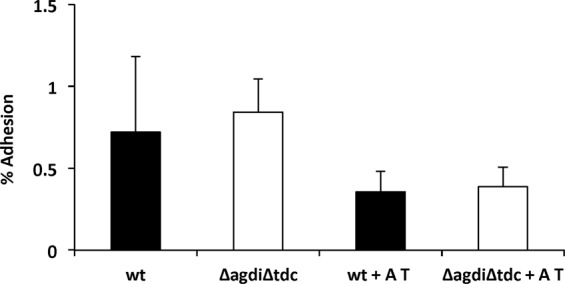
Adhesion of *E. faecalis* V583 (wt, black bars) and *E. faecalis* V583 *ΔagdiΔtdc* (Δ*agdi*Δ*tdc*, white bars) to Caco-2 cells in the absence or presence of 20 mM agmatine (A) and 10 mM tyrosine (T) after 5 h of co-culture. The adhesion level is expressed as a percentage of total bacteria after 5 h co-culture with Caco-2 cells in DMEM.

Figure 3 shows that 0.80% of both the wt and mutant bacterial cells adhered to the Caco-2 intestinal cells when agmatine and tyrosine were absent. In their presence, this figure was reduced to about 0.50% for both strains. During its incubation with the Caco-2 cells the wt strain produced 0.19 mM of putrescine and 0.14 mM of tyramine (as determined by UHPLC).

No BA was produced by the Caco-2 cells. These findings suggest that the intestinal adhesion capacity of the Δ*agdi*Δ*tdc* strain is similar to that of the wt strain.

### *E. faecalis* Δ*agdi*Δ*tdc* is able to form biofilms

**Figure 4:**
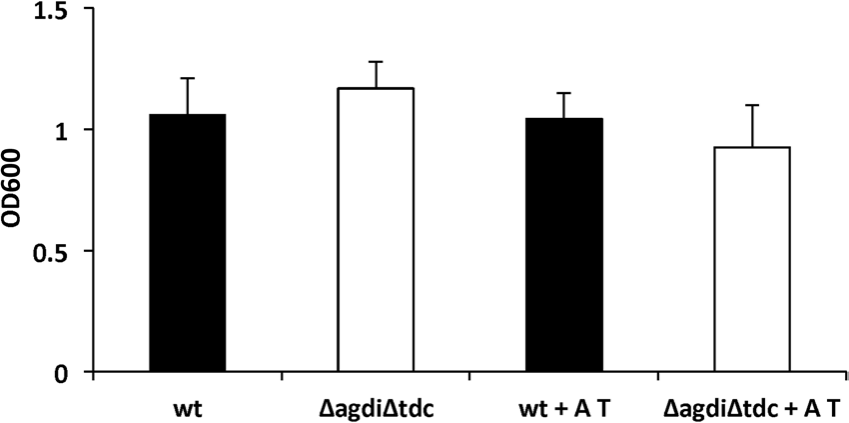
Biofilm formation by *E. faecalis* V583 (wt, black bars) and *E. faecalis* V583 *ΔagdiΔtdc* (Δ*agdi*Δ*tdc*, white bars) on polystyrene microtitre plates in GM17 in the absence or presence of 20 mM agmatine (A) and 10 mM tyrosine (T) after 16 h. Biofilms were assessed by crystal violet staining and the data corrected for the OD_600_.

To further confirm that BA production had no role in the above-mentioned adhesion, the ability of the wt and mutant strains to form biofilms on polystyrene surfaces was examined. No significant differences were observed between the BA-producer and non-producer strains in the presence, or in the absence, of agmatine and tyrosine, revealing BA formation not to be involved in the biofilm formation capacity of *E. faecalis* (Fig. 4).

### The transcriptome of *E. faecalis* is unaffected by the deletion of the *tdc* and *agdi* clusters

To determine whether the deletion of the *tdc* and *agdi* clusters affected the expression of other genes, the transcriptomes of *E. faecalis* Δ*agdi*Δ*tdc* and wt grown in GM17 were compared using the DNA microarray.

The results showed no gene outside of the deleted clusters to be differentially expressed in the mutant strain (Table 1). Since there is some tyrosine but no agmatine in GM17, the wt might have been able to express the genes of the tyrosine decarboxylase pathway. As expected, the expression of the deleted genes *tyrS*, *tdcA* and *tdcP* was null in the mutant strain. However, the *nhaC-2* gene appeared to be slightly overexpressed. The same has been reported for *E. faecalis* Δ*tdc* and is a consequence of the construction of the mutant and the microarray’s design (Perez *et al*., 2016^36^): (i) the mutant strain was constructed keeping the promoter of the first gene of the cluster (*tyrS*) and the 3′ end of the last gene in the cluster (*nhaC*-2), and (ii) one of the two *nhaC*-2 gene probes designed for the array hybridizes with the 3′ end of the remaining region of *nhaC*-2. Thus, in the presence of tyrosine, a polar effect causes the apparent expression of *nhaC*-2 in the Δ*tdc* mutant.

**Table 1:** Genes expressed significantly different in the transcriptome of Δ*agdi*Δ*tdc* compared to wt after 6 h of culture in GM17.

Gene	Locus	Function	Fold change	p-value
*tyrS*	EF0633	Tyrosyl-tRNA synthetase	−93.44	0.014
*tdcA*	EF0634	Tyrosine decarboxylase	−9.78	0.016
*tdcP*	EF0635	Tyrosine/tyramine exchanger	−4.32	0.020
*nhac-*2	EF0636	Na^+^/H^+^ antiporter	27.41	0.026

## Discussion

Allowing the presence of *E. faecalis* in food is controversial: some strains are used in the fermentation of foods^2^ and even as probiotics^3^, yet pathogenic strains responsible for serious nosocomial infections also exist^5^. An undesirable characteristic of this species is that it produces the biogenic amines tyramine and putrescine^13^, the accumulation of which in fermented foods can pose a risk to health^9^. In this work, a double mutant was constructed in which the clusters of genes responsible for the biosynthesis of these BA (*tdc* and *agdi* respectively) were absent. Its performance suggests the method followed might be appropriate for designing strains with desired technological properties but which are safer for consumers.

The similarity of the *agdi* cluster in the wt strain to those of others previously reported, and the inability of the Δ*agdi* mutant to produce putrescine in the presence of agmatine, confirm this cluster to be responsible for the biosynthesis of this BA. The involvement of the *tdc* cluster in tyramine production and its physiological role in protection against acid stress, were already known^16^.

Since the decarboxylation of amino acids consumes protons, this reaction affords a mechanism of resistance to acid stress in prokaryotes^22^. Moreover, the BAs thus synthesized, which possess a more positive charge than their precursor amino acids, are secreted from the cell, causing the net displacement of positive charges towards the exterior. This generates a proton-motive force that could be used by the cell to generate ATP via F1F0 ATPase^23,24^. Both functions have been demonstrated in *Enterococcus faecium*^25^. The decarboxylation of amino acids thus affords an advantage to microorganisms that face acidic environments such as those that occur in the stomach or in fermented foods.

The AGDI route has also been linked to acid stress resistance via the production of NH_3_. This also generates ATP, which could be used for the expulsion of protons from the cytoplasm via F1F0 ATPase^22^. In the LAB *Lactococcus lactis*, the AGDI route has been shown to enlist agmatine as a source of energy and to provide resistance to acid stress, counteracting the acidification of the cytosol^21,26^.

The present results obtained with the *E. faecalis* Δ*agdi*Δ*tdc* mutant show that it grows and resists acidic pHs less well than the wt strain (Fig. 1), but that it does survive acidic pH both in broth and under simulated gastric conditions (Figs 1 and 2). This is not very surprising, since the BA-producing pathways are only a piece of the acid stress resistance mechanisms. Indeed, it survived the conditions it would meet during GIT transit, with live bacteria reaching the colon in numbers similar to those seen for the wt strain (Fig. 2), and without their adhesion capacity (Fig. 3) or ability to form biofilms on polystyrene affected (Fig. 4). The influence of BA on cellular adhesion has been previously analysed in other BA-producing LAB strains having obtained different results. While the presence of tyrosine increases the adhesion of *E. durans* IPLA655 to Caco-2 cells^27^, it was not detected any influence of the tyramine and putrescine BA biosynthetic pathways on *L. brevis* adhesion capability^28^.

Importantly, the deletion of the clusters did not affect the expression of any non-related gene (Table 1), including any genes encoding pathogenicity factors. It should be remembered that the model strain used, *E. faecalis* V583, has several pathogenicity genes^29^, the expression of which could potentially have been affected. Thus, the gene cluster inactivation strategy followed could be used to construct *E. faecalis* strains with good technological characteristics but that do not produce BA, and perhaps even strains with good probiotic potential.

In the latest EFSA risk analysis of BAs, one of the strategies proposed to reduce BA concentrations in fermented food was the use of starter cultures with no BA-producing capacity (EFSA, 2011). Although this strategy could be used for those starter species in which the production of BA is a strain-dependent trait, in the case of *E. faecalis*, tyramine and putrescine production are species-dependent traits (Ladero *et al*., 2012^13^); this type of screening is therefore unviable. One alternative is the screening of spontaneous or induced mutants that have lost both characters - a tedious business requiring a large collection of mutants and with no guarantee of success. The present mutant construction strategy, however, does not suffer so strongly from these drawbacks.

In conclusion, the deletion of the *agdi* and *tdc* clusters gave rise to a strain of *E. faecalis* that does not produce the BAs putrescine and tyramine. Although the resulting mutant strain, *E. faecalis* Δ*agdi*Δ*tdc*, grew more poorly in the presence of agmatine and tyrosine than the wt strain, it survived *in vitro* GIT-like conditions. Importantly, the expression of no gene outside the removed clusters was affected. Taking into account (i) that the production of tyramine and putrescine is a species-level characteristic in *E. faecalis*^13^, (ii) that these BA have toxic and genotoxic *in vitro* effects^10,12^, and (iii) the results of this work, the double mutant approach would allow to obtain safer probiotic and starter *E. faecalis* strains.

## Materials and Methods

### Bacterial strains

The wild-type strain *E. faecalis* V583 (hereafter referred to as ‘wt’) was used as a model bacterium since its entire genome sequence is known. The strain was obtained from the American Type Culture Collection (accession number ATCC 700802). For all fermentation assays, overnight cultures of *E. faecalis* strains were used (0.1% *v*/*v* inoculum).

*Escherichia coli* Gene-Hogs (Invitrogen, UK) was used as an intermediate host for cloning^30^ in the construction of the knock-out mutant.

### DNA extraction

The GenElute Bacterial Genomic DNA Kit (Sigma-Aldrich, Spain) was used to extract total DNA from 2 mL of overnight cultures, following the manufacturer’s instructions. Plasmid extraction was performed following standard procedures^31^.

### PCR reaction and DNA sequencing

PCR reactions were performed in 25 µL reaction volumes with 1 µL of DNA as a template (typically 200 ng), 400 nM of each primer, 200 µM of dNTP (GE Healthcare, UK), the reaction buffer, and 1 U of Taq polymerase (Phusion High-Fidelity DNA Polymerase, Thermo Scientific, Spain). Reactions were performed in a MyCycler device (Bio-Rad, CA) with the program: 94 °C for 5 min, 35 cycles of 94 °C for 30 s, 55 °C for 45 s, 72 °C for 1 min, and a final extension step at 72 °C for 5 min. Table 2 shows the primers used (all synthesized by Macrogen [Seoul, Korea]). Primers were designed based on the *E. faecalis* V583 genome sequence (GenBank number: AE016830). PCR fragments were purified using the GenElute PCR Clean-Up Kit (Sigma-Aldrich) when needed. Sequencing of the PCR amplicons was performed at Macrogen.

**Table 2:** Primers used in this work.

Primer	Function	Sequence (5′-3′)	Reference
P1F	Deletion *agdi*	CTGCACCGACCATTATCTTATACTATGAAGGAA	This work
P2R	Deletion *agdi*	ATCGCCGTCTTCTCGCTGGCATGGTTTATTGGTGGGCTAAGCATTGGTTTCGGTGT	This work
P3R	Deletion *agdi*	AACCATGCCACGAGAAGACGGCGAT	This work
P4F	Deletion *agdi*	CATCAACTGTTTGGCTGTTTCTTCGTCATAATAAC	This work
AguR1F	*agdi* deletion check	ACTCCCAAAAATGATCGTAAAAACATG	This work
KagV5R	*agdi* deletion check	CAAAACGACCGATGTCCTACTCTCTAACG	This work
CardF	*tdc* deletion check	GATGATAGTGTCTTGGCTGCTTTAAAGG	^13^
EF0637R	*tdc* deletion check	GACTCGCTTGTGAAGTTGTCGCTGCAG	^13^

### Construction of the *Enterococcus faecalis* Δ*agdi*Δ*tdc* mutant

*E. coli* was routinely cultured at 37 °C with aeration in Luria-Bertani medium (Green and Sambrook, 2012[31]) supplemented with 1 mg L^−1^ ampicillin (USB Corporation,USA) when necessary. A mutant strain of *E. faecalis* V583 with the *tdc* and *agdi* clusters deleted (rendering it a non-tyramine and non-putrescine producer) was obtained by two subsequent steps of double-crossover homologous recombination using the cloning vector pAS222 as previously described^30^. The deletion of the *agdi* cluster was completed first. Sequence overlap extension PCR (SOE-PCR)^32^ was used to amplify the flanking fragments of the cluster. Two PCR reactions were performed with the primers P1 F and P2 R, and P3 F and P4 R (Table 2). The fragments were purified and then mixed to be used as the template for PCR amplification with the outer primers P1 F and P4 F. P3 R, the inner primer, carried regions of homology necessary for the fusion step. The amplicon was cloned into the *Sna*BI (Fermentas, Lithuania) site of pAS222 to generate pAS222 AGDI, which was propagated in *E. coli* Gene-Hogs cells. Electrocompetent *E. faecalis* V583 cells^16^ were transformed with pAS222 AGDI and the cells harbouring the plasmid grown in GM17 under previously described conditions to allow double-crossover recombination^33^. The formation of the intermediate mutant *E. faecalis* V583 with the deleted *agdi* cluster (hereafter referred to as ‘Δ*agdi*’) was confirmed by PCR with the primers AguR1F and KagV5R and amplicon sequencing, and by checking the lack of putrescine accumulation in overnight cultures in GM17 supplemented with 20 mM agmatine.

To effect *tdc* cluster deletion, electrocompetent cells of Δ*agdi* were produced and transformed with the plasmid pAS222 TDC, previously obtained by the same technique^16^. After double-crossover recombination, the deletion of the *tdc* cluster was confirmed via PCR with the primers CardF and EF0637R and further sequencing. The deletion of the *tdc* cluster encompassed the interval from *tyrS* (793 nt downstream of its start codon) to *nhaC-2* (691 nt downstream of its start codon), and *agdi* cluster deletion covered the interval from *aguR* (469 nt downstream of its stop codon) to *aguA* (785 nt downstream of its start codon). The confirmed deletion mutant *E. faecalis* V583 Δ*agdi*Δ*tdc* (hereafter referred to as ‘Δ*agdi*Δ*tdc*’) was used in further analyses.

### Growth of the wt and mutant strains under different conditions

*E. faecalis* V583 and the derived mutant *Δagdi*Δ*tdc* were grown at 32 °C without aeration in M17 (Oxoid, UK) supplemented 5 g L^−1^ glucose (Merck, Germany) (GM17). To determine the effect of the carbon source concentration, the same medium was supplemented with 1 g L^−1^ glucose. The influence of acidic pH was analyzed in GM17 adjusted to an initial pH of 5.0. Whenever necessary, media were supplemented with 20 mM agmatine and 10 mM tyrosine (Sigma-Aldrich). The optical density at 600 nm (OD_600_) was monitored over 10 h. For all the experiments, overnight cultures of *E. faecalis* strains were used (0.1% v/v inoculum).

### Resistance to gastrointestinal conditions

The assay described by Fernández de Palencia, *et al*.^34^, with the modifications of Perez, *et al*.^16^, was followed for the simulation of bacterial transit through the GIT. Briefly, approximately 10^10^ cfu mL^−1^ of the wt and Δ*agdi*Δ*tdc* strains from late exponential phase cultures (in GM17 supplemented with 20 mM agmatine and 10 mM tyrosine) were collected and mixed with the electrolyte solution (supplemented with the same concentrations of substrates). Cells were exposed first to lysozyme and then pepsin plus a successive reduction in pH to simulate gastric stress conditions. Gastrointestinal stress was mimicked by exposure of samples incubated at pH 5, 4.1, 3.0, 2.1 and 1.8 (gastric conditions), followed by their incubation in the presence of bile salts and pancreatin at pH 8 (small intestine conditions, GI). Colonic stress was simulated with the sample originally at pH 3 adjusted to pH 7 and incubated overnight. For each condition, cell viability was measured using the LIVE/DEAD^®^ BacLight fluorescent stain (Molecular Probes, Netherlands) as previously described^34^. The values provided are the mean of three independent replicates, expressed as a percentage of the untreated control. BA accumulation at the end of the assay was quantified as described below.

### Adhesion to the intestinal epithelium

The adhesion of the strains to the intestinal epithelium was studied in an *in vitro* model with Caco-2 cells obtained from the human cell bank at the *Centro de Investigaciones Biológicas* (Madrid, Spain), following the protocol previously described ^34^ with minor modifications. These cells were grown in Men-Alpha Medium (Invitrogen) supplemented with 10% (v/v) heat-inactivated foetal bovine serum at 37 °C in a 5% CO_2_ atmosphere, and then seeded in 24-well tissue culture plates (Falcon Microtest™, Becton Dickinson, USA) at 4 × 10^4^ cells per well, and grown for 15 days to obtain a monolayer of differentiated, polarized cells. The bacterial strains were grown in GM17 supplemented with 20 mM agmatine and 10 mM tyrosine until the end of the exponential phase. They were then washed with phosphate buffered saline pH 7.1 (PBS) and resuspended in Dulbecco’s Modified Eagle medium (DMEM) (Invitrogen). The Caco-2 cells and bacterial strains were then co-cultured at a ratio of 1:100 in DMEM in the presence or absence of 20 mM agmatine and 10 mM tyrosine. After 5 h of incubation at 37 °C in a 5% CO_2_ atmosphere, the cells were washed three times with 500 µL PBS and resuspended in 0.1 mL of PBS. Non-washed wells were used as a control. The Caco-2 cells were detached by the addition of 500 µL trypsin-EDTA (0.05%) (Gibco, USA) for 10 min at 37 °C; the reaction was inactivated by adding 500 µL PBS at 4 °C. The total number of bacteria was determined by serial dilutions and plate counts, and the number adhered to the intestinal cells calculated as a percentage of the total bacteria in the unwashed controls. Each adhesion assay was performed in triplicate. BAs produced at the end of the assay were detected as indicated below.

### Measurement of biofilm-forming capacity

The capacity of the strains to form a biofilm was tested on polystyrene microtitre plates (TC Microwell 96U, Thermo Scientific)^35^. Overnight cultures in GM17 with or without 20 mM agmatine and 10 mM tyrosine were diluted 1:40 in 200 µL of the same medium. After 16 h of incubation in the microtitre plates, the cells were washed, stained with crystal violet, and their OD_600_ determined.

### DNA microarrays and data analysis

Agilent eArray v5.0 software (Agilent Technologies, USA) was used to design a DNA microarray for *E. faecalis* V583. The array harboured duplicate spots of two different 60-mer probes specific for each of the 3182 coding DNA sequences (CDSs) in the chromosome of the above strain (GenBank accession no. AE016830)^29^. The microarray design was added to the Gene Expression Omnibus (GEO) database (Platform GPL21449). Total RNA was extracted from late exponential phase cultures of the wt and Δ*agdi*Δ*tdc* mutant grown in 30 ml of GM17 supplemented with 20 mM of agmatine and 10 mM of tyrosine as previously described^36,37^. 20 µg of RNA were used to synthesize cDNA employing the SuperScript III Reverse Transcriptase Kit (Life Technologies, The Netherlands). cDNA (20 mg) was labeled with DyLight 550 or DyLight 650 using the DyLight Amine-Reactive Dyes Kit (Thermo Scientific). The hybridization step was carried out with 900 ng each of DyLight 550- and DyLight 650-labeled cDNA for 17 h at 60 °C on the *E. faecalis* V583 DNA microarray using the *In situ* Hybridization Kit Plus, a hybridization gasket slide, and a G2534 A microarray hybridization chamber^36^, all from Agilent Technologies. Slides were scanned using a GenePix 4200 A Microarray Scanner (Molecular Devices, USA) and images acquired and analyzed using GenePix Pro v.6.0 software. The standard routines provided by GENOME2D software (http://genome2d.molgenrug.nl/index.php/analysispipeline) were used for background subtraction and locally weighted scatterplot normalization. Microarray data were obtained from two independent cultures and one technical replicate that included a dye swap. Expression ratios were calculated from the comparison of four spots per gene per microarray.

Genes returning a significant difference (*p* ≤ 0.05) in their wt and mutant expressions, plus an expression fold-change of at least double, were considered differentially expressed. The microarray data is available in the GEO database under the Accession no. GSE136953. Functional analysis was executed using Gene Set Enrichment Analysis (GSEA) routines as provided by GENOME2D (http://server.molgenrug.nl/index.php/gsea-pro).

### Quantification of biogenic amine synthesis

Samples obtained during the above experiments were centrifuged and the supernatants recovered and filtered through 0.45 µm polytetrafluoroethylene (PTFE) filters (VWR, Spain), The detection and quantification of the BAs and their substrates was performed by UHPLC. The compounds were derivatized with diethyl ethoxymethylenemalonate (Sigma-Aldrich) and separated in a UPLC system (Waters, USA) under the conditions described ^38^. Chromatograms were obtained and analysed using Empower 2 software (Waters). The results are the means at least three replicates.

### Statistical analysis

Data are presented as the means ± standard deviations calculated from at least three independent replicates. Means were compared by the Student *t* test using SPSS software v.15.0 (SPSS, Inc., USA). Significance was set at *p* ≤ 0.05.
